# The movement profile of trunk and neck during habitual vacuuming

**DOI:** 10.1038/s41598-021-99664-4

**Published:** 2021-10-14

**Authors:** C. Maurer-Grubinger, J. Haenel, L. Fraeulin, F. Holzgreve, E. M. Wanke, D. A. Groneberg, D. Ohlendorf

**Affiliations:** Center of for Health Sciences, Institute of Occupational, Social and Environmental Medicine, Goethe-University Frankfurt Am Main, Theodor-Stern-Kai 7, Building 9B, 60590 Frankfurt am Main, Germany

**Keywords:** Occupational health, Musculoskeletal system, Preventive medicine

## Abstract

Musculoskeletal disorders of the trunk and neck are common among cleaners. Vacuum cleaning is a demanding activity. The aim of this study was to present the movement profile of the trunk and neck during habitual vacuuming. The data were collected from 31 subjects (21f./10 m) using a 3D motion analysis system (Xsens). 10 cycles were analysed in vacuuming PVC and carpet floors with 8 vacuum cleaners. The joint angles and velocities were represented statistically descriptive. When vacuuming, the trunk is held in a forwardly inclined position by a flexion in the hip and rotated from this position. In the joint angles and velocities of the spine, the rotation proved to be dominant. A relatively large amount of movement took place in the cervical spine and also in the lumbar spine. The shown movement profile is rather a comfort area of vacuuming which may serve as a reference for ergonomics in vacuuming.

## Introduction

Musculoskeletal complaints are a highly relevant health problem in the working population, which are the most frequent cause of incapacity to work in Germany, accounting for almost 25% of all cases^1,2^. The most common diagnosis is back pain^1^. One of the occupational groups with the highest number of days of incapacity to work due to musculoskeletal complaints were cleaners^1^. Several research have shown that the back (especially the lower back and neck) is frequently stressed during cleaning work and is characterized as a frequent area of discomfort^3–6^. In the 12-month prevalence, 45.5% of urban cleaners in Brazil experienced pain in the spinal region, 15.7% in the neck and 14.3% in the upper back^7^. Musculoskeletal discomfort among cleaners in Taiwan was 37.8% in the lower back, 12.8% in the neck, and 3.3% in the upper back^3^. The prevalence of musculoskeletal pain and discomfort among cleaners in the UK was even higher with 74% in the 12-month prevalence and 52% in the 7-day prevalence^6^. The lower back was affected in 46% (12-month prevalence) and 24% (7-day prevalence) of the cleaners and the neck in 33% (12-month prevalence) and 19% (7-day prevalence) of the cleaners^6^. Many cleaners regarded work activity as a suspected cause of musculoskeletal complaints^3,5,6^. Among the suspected causal activities buffing, mopping and vacuuming are mentioned^5,6^. Similarly, mopping (17.9%) and vacuuming (12.3%) were the most frequently mentioned activities that subjectively lead to fatigue^8^.

The literature indicates the following risk factors for the musculoskeletal system during physically demanding activities, such as cleaning: pushing and pulling or repetitive work^4,9–12^, awkward postures (in bent and rotated positions)^4,6,7,13^, and static muscle work^4^. Repeated pushing and pulling can cause repeated stress on the tissue, which can lead to tissue injuries, especially with high forces^9,10^. However, pushing and pulling does not appear to be a significant work-related risk factor since the evidence concerning the relationship of these movements and low back pain is weak or inconsistent^10,12^. Nevertheless, pushing and pulling or repetitive work in cleaning activities seems to be a relevant physical stress factor. Pekkarinen^11^ showed that cleaners found "moving continuously from place to place" and "monotonous repetitive work movements" to be the greatest physical stress factors. Marut and Hedge^8^ cited moving back and forth while vacuuming as one reason why this household activity was perceived as tiring. The circumstances under which repetitive work leads to fatigue also seem to depend on the posture adopted during this activity. Non-neutral body postures can increase physical strength requirements and thus the potential risk of musculoskeletal complaints^4,9^. In their systematic review, Wai et al.^13^ refer to a weak to moderate evidence concerning a causal relationship between occupational bending or twisting and low back pain. Among awkward postures observed in cleaners were back positions in rotation and flexion and a static neck flexion^6^. Pataro and Fernandes^7^ identified combinations of extreme rotational and flexion postures as a risk of low back pain in urban cleaners. When vacuuming as cleaning activity, bent, multidimensionally twisted, awkward body postures and movements are described^5,8,11^. Frefer et al.^14^ investigated two vacuum cleaner models under different trunk flexion angles, according to which the trunk flexion angle was more relevant than the vacuum cleaner type in terms of the effect on muscle activity and the perception of discomfort. Awkward postures can be particularly problematic if they are maintained static. Cleaners often report awkward and/or static postures of the back and arms^4^. However, dynamic works tend to have a protective effect. Thus, activities carried out with low force and high repetition can even reduce the risk of musculoskeletal complaints^9^. Pataro and Fernandes^7^ identified dynamic working as a protective factor in relation to low back pain in urban cleaners.

Single factors, such as pushing and pulling or bending and twisting, do not seem to be independent factors in causing work-related musculoskeletal complaints^12,13^. Rather these complaints seemed to be multifactorial. Thus, various factors play a role in the development of musculoskeletal complaints, such as repetitive work, the necessary expenditure of strength, awkward postures or the loading duration^4,9^. When vacuuming, a summation of risk factors is conceivable, as this activity represents repetitive pushing and pulling using the trunk and the upper extremity. The back and neck appear to be vulnerable areas during cleaning, which can be endangered by the mentioned risk factors. The summation of risk factors such as the repetitive movement in non-neutral body postures during vacuuming leads to musculoskeletal disorders. The back and the neck are under strain during this task. Thus, the aim of this study was to investigate joint angles of the trunk and neck during habitual vacuuming in order to generate initial reference values. The objectives of this study were (a) to describe the basic movement profile of the neck, trunk, and hip joints during habitual vacuuming on a vacant surface and (b) to identify potential musculoskeletal risk factors associated with cleaning work in the vacuuming cycle: pushing and pulling or repetitive work, awkward body positions (bent, flexed and rotated) and static work sequences.

## Materials and methods

### Subjects

The sample size calculation was based on preliminary measurements. It was assumed that the average maximum trunk flexion angle is about 37°. The associated standard deviation is estimated at a maximum of about 6°. With a number of 30 subjects, the mean value can be statistically validated to 4.5°. This means that the length of the corresponding 95% confidence interval is about 4.5° with this number of subjects. A gender ratio of 1/3 men and 2/3 women was aimed for in subject recruitment, as women spend more time on household activities than men^15^. Women therefore seem to be more predisposed to the provocation of musculoskeletal complaints by household activities^16^. The targeted gender ratio should reflect the gender difference in household activities.

31 healthy subjects (21f./10 m; age: 33.4 ± 10.7 years, height: 172.8 ± 9.4 cm; mass: 66.9 ± 13.9 kg) participated in this study. Participants, consisting of healthy and non-professional cleaners, were recruited by a convenience sampling from the surrounding area of the Goethe University Frankfurt am Main. The handedness was asked by a questionnaire. One subject was left-handed, the remaining 30 subjects were right-handed. A contraindication questionnaire was used to inquire the medical history of the subjects. Especially it was checked if subjects have been recently or are currently in medical therapy and if they suffer on pain during cleaning activities. Subjects who had a diagnosed injury were excluded from the study. Especially the exclusion criteria for the study were current injuries (herniated discs, spinal column injuries), rheumatic diseases, severely restrictive spinal deformities (scoliosis) or stiffened spinal column joints (pathological or surgical) and genetic muscle diseases. A positive ethics approval from the ethics committee of the Department of Medicine of the Goethe University Frankfurt am Main (ethic-number: 335/18) was obtained. All subjects gave written informed consent before participating within the study. All methods were performed in accordance with relevant guidelines and regulations.

### Measurement system

An inertial sensor based motion capture system (Xsens, Enschede, Netherlands) was used to collect the 3D motion. The system records the linear acceleration and angular rotation of the musculoskeletal system with 17 sensors. The sensors are tightly attached to the body on well-defined positions (lower and upper extremities, trunk and head). The sensors were positioned on the top of the feet (left and right), at the shank (left and right), thigh (left and right), pelvis, at the spine in the height of the Th8, on the head (wristband), on the acromion (left and right), upper arm (left and right), lower arm (left and right) and the dorsum of the hand (left and right, using a glove). The calibration procedure of the sensors was as following. First subjects stood in an upright position, arms straight beside the body facing to the floor, the head in a neutral position with eyes pointing forward. Body and legs were positioned in a neutral position as well. A trained physiotherapist controlled the neutral position. Second, the subjects walked five steps forward, made a 180° turn and came back to the standing position. Validity studies show, that sensor based systems show good-to-excellent results compared to optical motion capture systems especially the sagittal and frontal planes^17,18^. The manufactures software MVN Analyse was used to calculate joint angles and the velocities of the segments. The sampling rate was set to the highest available rate of 240 Hz. The manufacturer specified the measurement error with ± 1%.

### Vacuum cleaners

All used vacuum cleaners were household cleaners where the floor could be cleaned in upright positions. Eight cleaners were selected to somehow cover the wide range of available vacuum cleaners. The cleaners differed especially in the location of the canister. Four vacuum handstick cleaners were selected, where the canister is integrated in the style and pipe. In addition, four-cylinder vacuum cleaners were chosen, where the hose is connected to a canister standing on the floor. All vacuum cleaners differ in dimensions, weight and adjustment controls. The specific properties of the eight domestic vacuum cleaners are summarized in Table 1.

**Table 1:** Properties of the vacuum cleaners according to the manufacturers' specifications.

	Handstick vacuum cleaners				Cylinder vacuum cleaners			
	No.1	No.2	No.3	No.4	No.5	No.6	No.7	No.8
Length (cm)	18.2	55.4	85–109	24.0	49.6	38.3	43.0	29.8
Width (cm)	26.1	/	21	25.0	/	37.0	33.0	26.4
Height min.–max. (cm)	62.1–123.4	16.5	15	125.0	22.7	37.3	26.0	52.8
Weight (N)	32	50.2	30	29	72.6	66	60	79
Adjustable handle	Yes	Yes	Yes	no	Yes	Yes	No	Yes

### Measuring protocol

A standardized habitual vacuum motion was recorded. Prior to the measurement, the subjects adjusted the handle of the vacuum cleaner to their preferred length. The motion was defined as a cyclic movement in an upright position, with both feet on the ground. The task was to push the cleaner forward and pull it back. This task was twelve times repeated. The feet were placed in a step position, with the contralateral leg to the dominant hand in front. The vacuuming movement was performed in the viewing direction in a habitual manner, meaning they could either use both or only one hand. Two different floor conditions were recorded: PVC floor and on carpet.

From the twelve movement cycles the first and last were removed. The remaining 10 cycles were cut based on the position of the hand marker in the movement direction. Every step cycle started and ended at the distal turning point. The proximal inflection point was used to calculate the duration of the back-and-forth movement. The pulling (back) movement started at the distal turning point and ended at the proximal turning point, the pushing (forward) movement started at the proximal turning point and lasted until the distal turning point.

### Data processing

Joint angles were exported from the MVN analyzer provided by the manufacturer. All further analysis steps were done in costume written scripts in MATLAB vR2018a software (The Mathworks Inc., Natick, MA, USA). The focus of this study was set on the trunk motion. Therefore, the joint angles related to the spine and the two hip joints were used for further analysis. The output of the manufactures human body model for the spine are the joints between Head and C1, C7–T1, T8–T9, T12–L1, L3–L4, L5–S1. The later four (T8–T9, T12–L1, L3–L4, L5–S1) had comparable movement patterns. Therefore, the cumulative sum was presented (Fig. 2). The vacuum cycle was time normalized to 100 time steps. We have followed a dominant side protocol. Therefore, we have adjusted the joint angles of the left-handed subject to align with the joints of the right-handed subjects. Manual inspected of the data revealed that some movement cycles were corrupted. Because of the amount of individual traces (119,040 (10 cycles × 2 floor conditions × 8 cleaners × 31 subjects × 8 joints × 3 dimensions)) an automatized procedure was used. Individual cycles were compared against the range of the entire data pool. Cycles, that were outside a range of mean ± 5 SD were removed from the sample. If more than 3 outliers were detected within a joint movement, all trials of the specific subject were removed from the sample. In total 13 trials had to be removed, that is 0.3% of all trials.

### Statistical analysis

**Figure 1:**
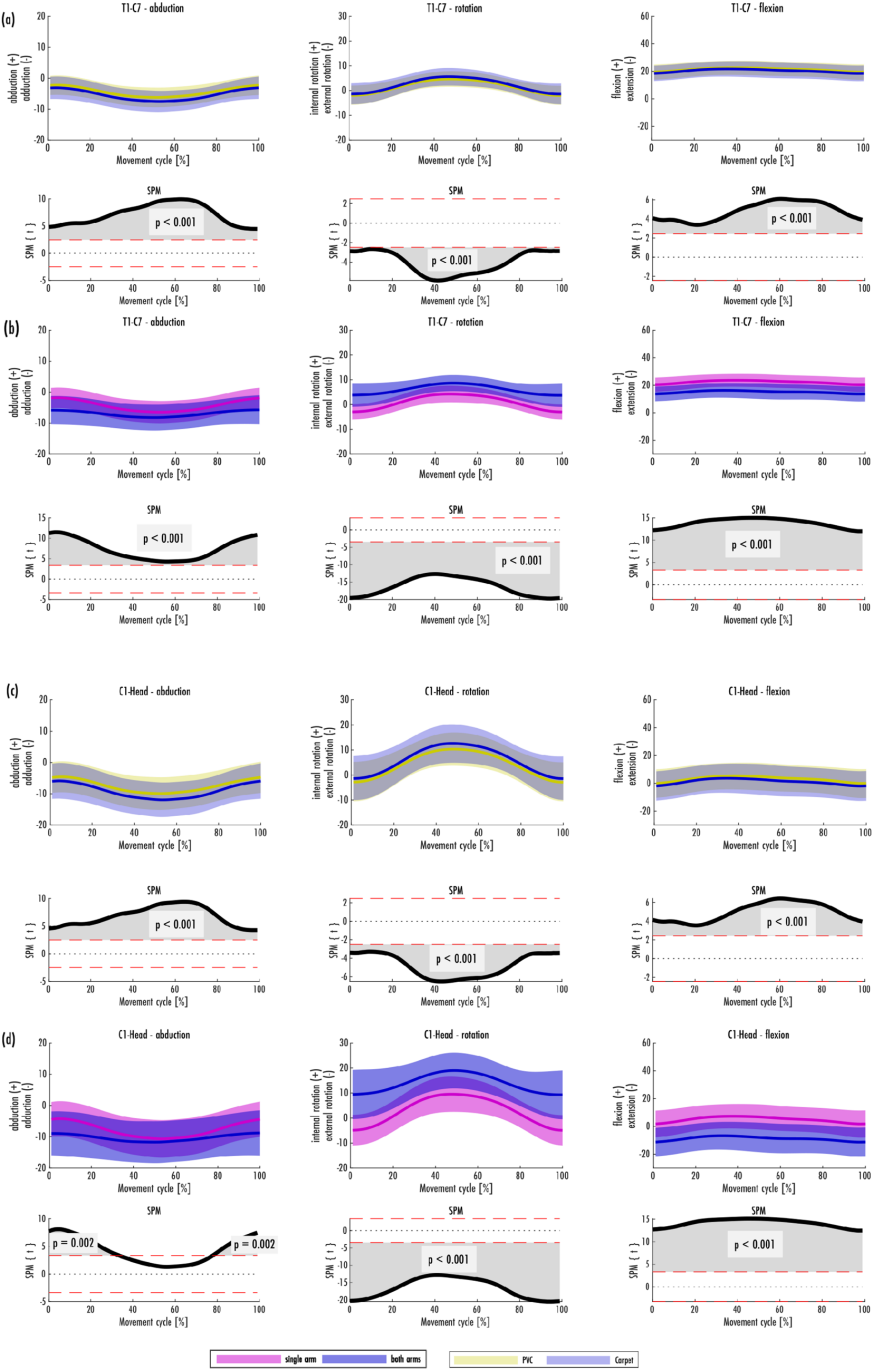
Spinal movements in the areas C1-Head (**a**, **b**) and T1-C7 (**c**, **d**) in the habitual vacuuming cycle [mean and range of standard deviation (± SD)]. (**a**, **c**) are grouped based on the carpet condition, (**b**, **d**) are grouped based on the grip position. The result of the SPM is plotted directly underneath the mean and SD of the groupings.

**Figure 2:**
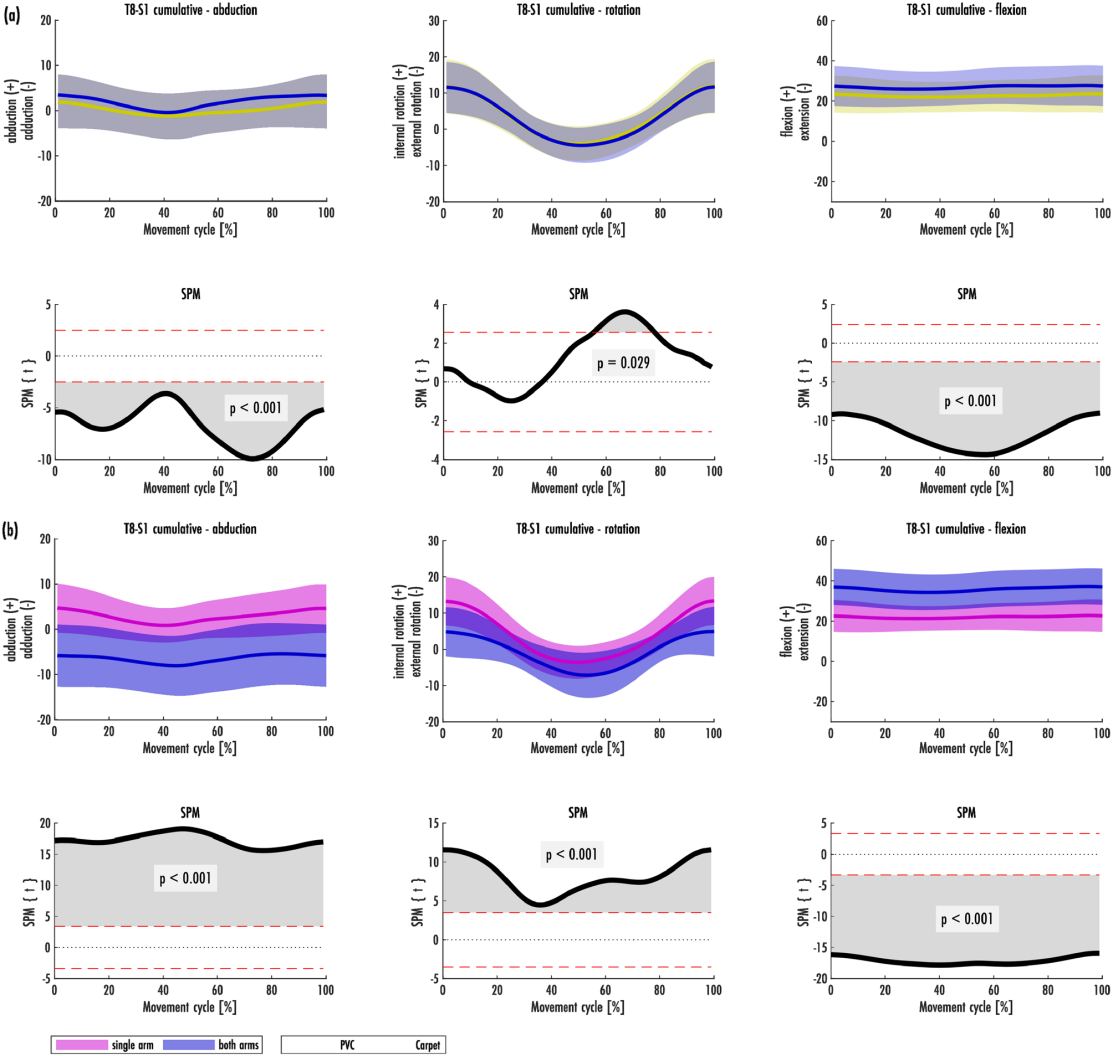
**a**, **b** Spinal movements of the T8-S1 cumulative joint T8–S1 in the habitual vacuuming cycle [mean and range of standard deviation (± SD)]. **a** Is group based on the carpet condition, **b** is the group based on the grip position. The result of the SPM is plotted directly underneath the mean and SD of the groupings.

**Figure 3:**
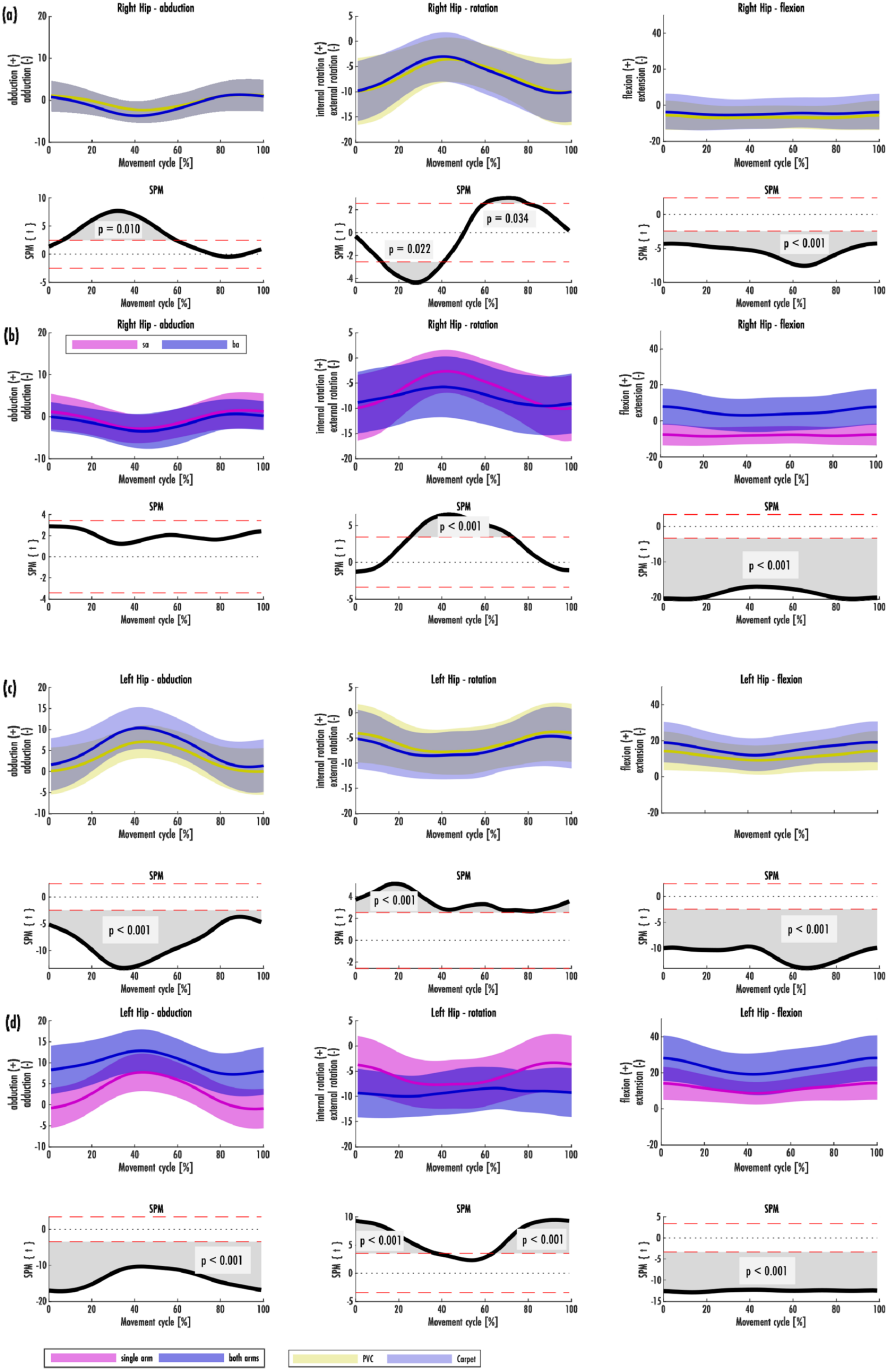
Hip joint movements right (**a**, **b**) and left (**c**, **d**) in the habitual vacuuming cycle [Mean and range of standard deviation (± SD)]. (**a**, **c**) are grouped based on the carpet condition, (**b**, **d**) are grouped based on the grip position. The result of the SPM is plotted directly underneath the mean and SD of the groupings.

Means and standard deviations (SD) were calculated for the vacuuming cycle distance and duration. Means and SDs for the joint dimensions were calculated at cycle start/end (distal turning point) and at mid-cycle (proximal turning point). Furthermore, the maximum and minimum of mean movement (mean values over the 100 normalized points in time) with its SD and its relative point in time in the vacuuming cycle were determined. The mean SD over the whole cycle and its SD were also calculated (see Table 2). Joints were represented in their movement dimensions by the mean movement and the range of standard deviation (± SD) over the cycle (Figs. 1a, b, 2a, b, 3a, b).

**Table 2:** Mean values and standard deviations (SD) of movements of C1-Head, T1-C7, T8-S1 cumulative, right and left hip over the course of the cycle.

Joints	Dimensions	Mean ± SD at cycle start/end (distal turning point) in degree (°)	Mean ± SD at mid-cycle (proximal turning point) in degree (°)	Maximum mean ± SD in the cycle in degree (°) [normalized time unit in the movement cycle in%]	Minimum mean ± SD in the cycle in degree (°) [normalized time unit in the movement cycle in%]	Mean of SD ± SD (over the cycle) in degree (°)
C1-Head	Lateral bending right ( +)/Lateral bending left ( −)	− 5.39 ± 6.11	− 10.92 ± 6.13	− 5.22 ± 6.18 [5%]	− 10.95 ± 6.12 [53%]	6.03 ± 0.11
	Rotation left ( +)/rotation right ( −)	− 2.12 ± 8.91	11.41 ± 7.92	11.49 ± 7.96 [48%]	− 2.18 ± 8.84 [100%]	7.83 ± 0.58
	Flexion ( +)/extension ( −)	− 0.98 ± 10.95	3.77 ± 10.94	4.49 ± 10.52 [36%]	− 0.99 ± 11.07 [98%]	10.71 ± 0.20
T1-C7	Lateral bending right ( +)/Lateral bending left ( −)	− 2.73 ± 3.86	− 6.86 ± 3.76	− 2.66 ± 3.90 [4%]	− 6.87 ± 3.76 [52%]	3.72 ± 0.1
	Rotation left ( +)/rotation right ( −)	− 1.68 ± 4.32	5.08 ± 3.74	5.14 ± 3.76 [47%]	− 1.71 ± 4.296 [100%]	3.75 ± 0.3
	Flexion ( +)/extension ( −)	18.98 ± 5.82	21.81 ± 5.76	22.14 ± 5.57 [38%]	18.97 ± 5.88 [98%]	5.66 ± 0.11
T8-S1 cumulative	Lateral bending right ( +)/Lateral bending left ( −)	2.66 ± 7.09	− 0.20 ± 5.92	2.66 ± 7.09 [1%]	− 0.82 ± 5.70 [42%]	6.29 ± 0.44
	Rotation left ( +)/rotation right ( −)	11.70 ± 7.45	− 4.21 ± 5.09	11.81 ± 7.47 [100%]	− 4.21 ± 5.07 [50%]	5.63 ± 0.96
	Flexion ( +)/extension ( −)	25.42 ± 9.87	24.54 ± 8.58	25.57 ± 9.90 [95%]	23.95 ± 8.44 [33%]	9.14 ± 0.53
Right hip	Abduction ( +)/adduction ( −)	0.92 ± 4.11	− 2.70 ± 3.599	1.28 ± 4.19 [90%]	− 3.05 ± 3.58 [43%]	3.83 ± 0.22
	Internal rotation ( +)/external rotation ( −)	− 9.89 ± 6.51	− 3.92 ± 4.81	− 3.31 ± 4.81 [42%]	− 10.11 ± 6.34 [95%]	5.46 ± 0.63
	Flexion ( +)/extension ( −)	− 4.70 ± 9.21	− 5.79 ± 7.45	− 4.70 ± 9.21 [1%]	− 6.23 ± 7.60 [28%]	8.02 ± 0.65
Left hip	Abduction ( +)/adduction ( −)	0.84 ± 6.18	8.28 ± 4.89	8.72 ± 4.99 [44%]	0.54 ± 6.01 [95%]	5.26 ± 0.49
	Internal rotation ( +)/ external rotation ( −)	− 4.62 ± 6.12	− 7.95 ± 4.89	− 4.22 ± 6.09 [92%]	− 8.14 ± 4.72 [38%]	5.27 ± 0.53
	Flexion ( +)/extension ( −)	16.61 ± 11.24	10.99 ± 8.69	16.70 ± 11.26 [99%]	10.54 ± 8.58 [44%]	9.59 ± 0.97

**Figure 4:**
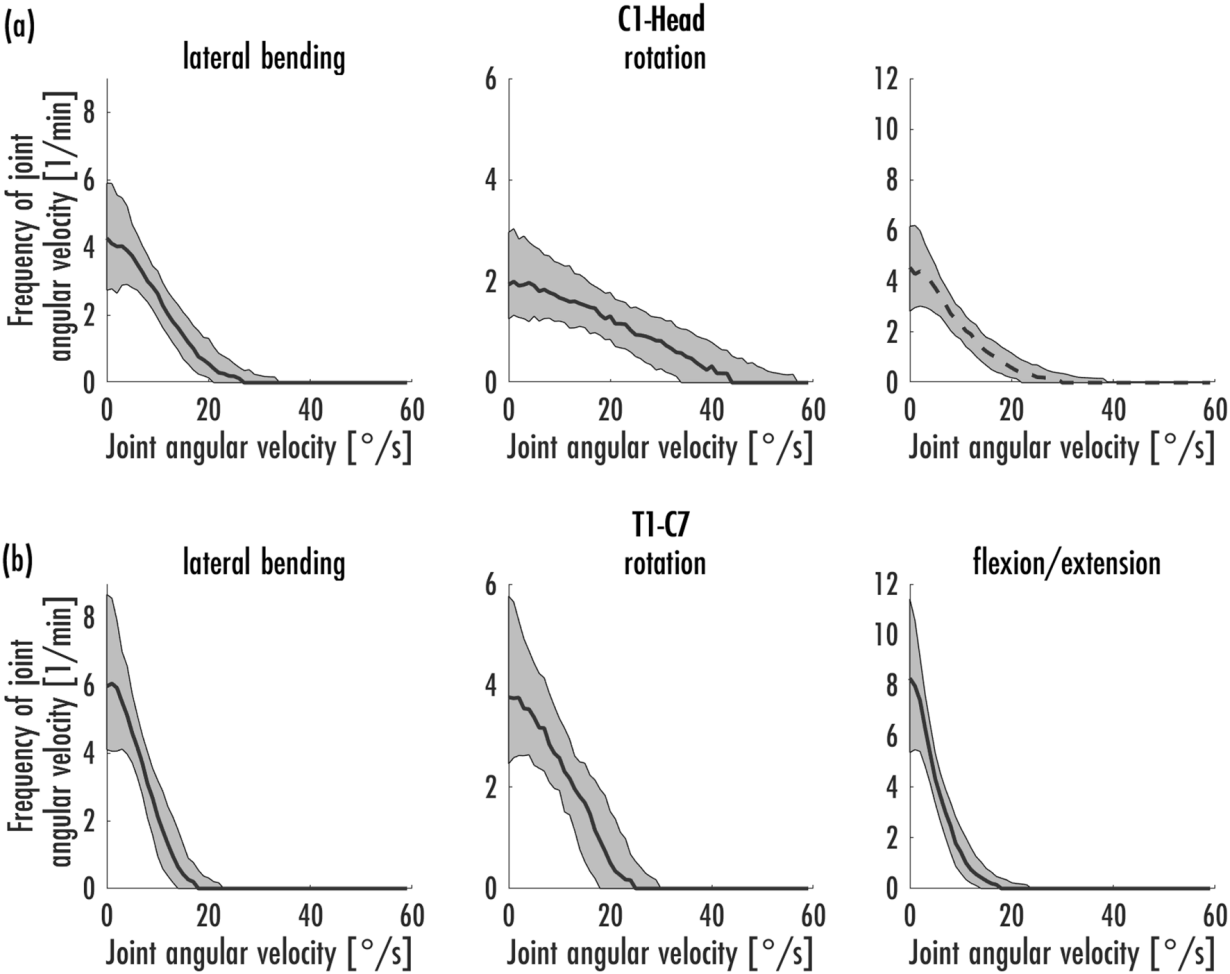
Frequencies of joint angular velocity [1/min] in the areas C1-Head (**a**) and T1-C7 (**b**) in habitual vacuuming [median, 25%- and 75%-percentiles].

**Figure 5:**
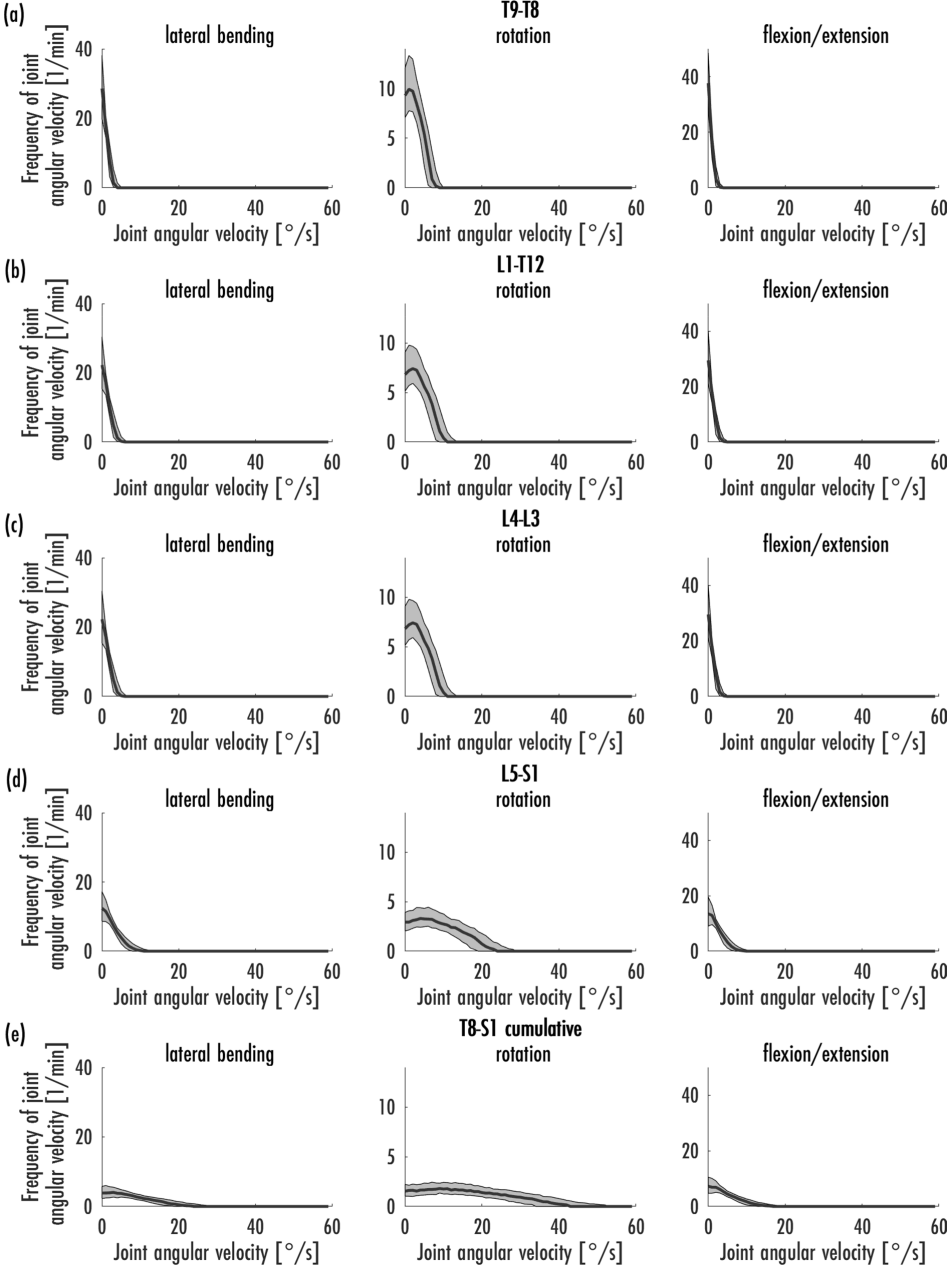
Frequencies of joint angular velocity [1/min] in the areas T9–T8 (**a**), L1-T12 (**b**), L4-L3 (**c**), L5-S1 (**d**) and T8-S1 cumulative (**e**) in habitual vacuuming [median, 25%- and 75%-percentiles].

**Figure 6:**
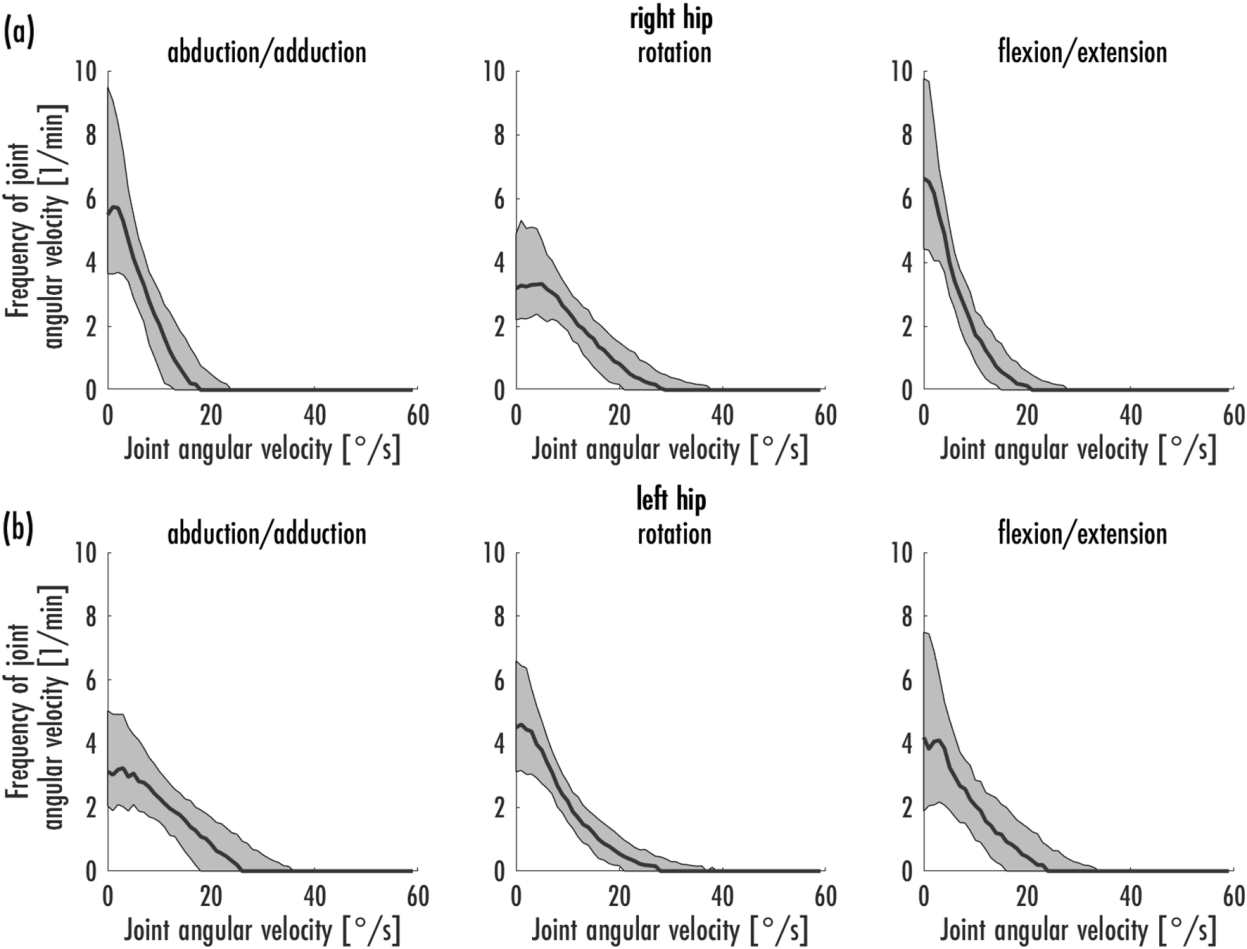
Frequencies of joint angular velocity [1/min] in hip joints right (**a**) and left (**b**) in habitual vacuuming [median, 25%- and 75%-percentiles].

The median and interquartile range (IQR) of the joint angle velocities (°/s) were calculated (Table 3). Furthermore, the mean maximum and minimum velocity was calculated in several steps. First, the maximum and minimum velocity per cycle and person was determined. Next, the mean values of the maximum and minimum velocities were calculated from the ten cycles of a condition. The calculation of the mean maximum and minimum velocity was performed by calculating the mean value from the previous calculations over the conditions and participants (Table 3). In addition, the frequencies of joint angular velocities within a minute were graphically represented by the median with the 25% and 75% percentiles (Figs. 4a, b, 5a–e, 6a, b). The frequency per minute was calculated by dividing the spectrum of a cycle by the time of that cycle.

**Table 3:** A high level summery of the significant differences between the two floor conditions and the two grip positions.

	Abduction	Rotation	Flexion
**PVC versus carpet**			
C1-Head	PVC higher abduction (*p* < 0.001)	PVC lower internal rotation (*p* < 0.001)	PVC higher flexion (*p* < 0.001)
T1-C7	PVC higher abduction (*p* < 0.001)	PVC lower internal rotation (*p* < 0.001)	PVC higher flexion (*p* < 0.001)
T8-S1 cumulative	PVC lower abduction (*p* < 0.001)		PVC lower flexion (*p* < 0.001)
Right Hip			PVC lower flexion (*p* < 0.001)
Left Hip	PVC lower abduction (*p* < 0.001)	PVC higher internal rotation (*p* < 0.001)	PVC lower flexion (*p* < 0.001)
**Single arm versus double arm**			
C1-Head	Single arm higher abduction (*p* < 0.001)	Single arm lower internal rotation (*p* < 0.001)	Single arm higher flexion (*p* < 0.001)
T1-C7	Single arm higher abduction (*p* < 0.001)	Single arm lower internal rotation (*p* < 0.001)	Single arm higher flexion (*p* < 0.001)
T8-S1 cumulative	Single arm higher abduction (*p* < 0.001)	Single arm higher internal rotation (*p* < 0.001)	Single arm lower flexion (*p* < 0.001)
Right Hip		Single arm higher internal rotation (*p* < 0.001)	Single arm lower flexion (*p* < 0.001)
Left Hip	Single arm lower abduction (*p* < 0.001)	Single arm higher internal rotation (*p* < 0.001)	Single arm lower flexion (*p* < 0.001)

In general, the difference between the floor condition is in the order of 2° to 3° while the difference between the holding position is between 5° and 10°.

Statistical significance was tested using statistical parametric mapping (SPM)^19^. With this method, the whole waveforms were analyzed. The alpha value of significance was set to α = 0.05. Because of the multiple joints and directions, a Bonferroni correction was applied. The correction factor was 15 (5 joints (as only the cumulative joint of the trunk was used) and 3 directions).

## Results

The distance of sections in the vacuuming cycle (pulling and pushing movement) averaged 0.65 ± 0.20 m (m). One whole vacuuming cycle lasted on average 1.65 ± 0.38 s (s).

### Motion profile: joint angles

Mean values and standard deviations of movements of the head/cervical spine, lower back and hip joints over the course of the cycle are shown in Table 2.

Figure 1a–d shows the movements of the head and cervical spine. Towards the middle of the cycle the head and cervical spine were increasingly tilted and rotated to the left. The amplitudes of lateral flexion and rotation to the left were more pronounced in the C1-Head joint than in the T1–C7 joint. Extreme values were seen at the beginning and end as well as in the middle of the cycle. The difference between the maximum and minimum mean in the C1-Head joint was 5.76° in lateral flexion (from 5.22° to 10.95° inclination to the left side) and 13.67° in rotation (from 2.18° right rotation to 11.49° left rotation), whereas in the T1-C7 joint it was 4.21° (from 2.66° to 6.87° inclination to the left side) and 6.85° (from 1.71° right rotation to 5.14° left rotation) respectively. The maximum flexion of the C1-Head and T1-C7 joints were at almost 40% of the cycle, while flexion was lowest at the end of the cycle. The difference between minimum and maximum mean was 5.48° in the C1-Head joint (from 0.99° extension to 4.49° flexion) and 3.17° in the T1-C7 joint (from 18.97° to 22.14° flexion).

Figure 2a, b shows the movements of the T8-S1 joints in a cumulative picture. Rotation in the trunk from left to right side was clearly pronounced, while lateral flexion (more to the right side) and flexion were more on the same level. The difference between the maximum and minimum mean was 16.02° for T8-S1 cumulative in rotation (from 11.81° left rotation to 4.21° right rotation), while the difference was 3.48° for lateral flexion (from 2.66° inclination to the right side to 0.82° to the left side) and 1.72° for flexion/extension (from 23.95° to 25.57° flexion). The trunk was more inclined to the right and moves near the middle of the cycle into a slight left lateral flexion. The rotational movement moved towards the middle of the cycle into an increasing rotation to the right, in contrast to the increased rotation to the left in the head and cervical spine. The trunk was kept in a flexion of approx. 25° over the entire cycle.

Figure 3a–d shows the movements of the hip joints. To the middle of the cycle the right hip was slightly adducted and the left hip was abducted by 8.28° ± 4.89°. The hip joints were externally rotated, with the external rotation of the right hip decreasing towards the middle of the cycle and that of the left hip increasing. The right hip was in a slight extension position throughout the cycle. The left hip was primarily flexed. The flexion decreased from the beginning/ end of the cycle to the middle of the cycle. The difference between the maximum and minimum mean in the flexion of the right hip was 6.16°.

The mean standard deviation over the entire cycle was greatest in all joints in the dimension of flexion/ extension compared to lateral flexion or abduction/ adduction and rotation. The largest mean standard deviations were observed in the C1-Head joint and the smallest in the T1-C7 joint.

A summary of all the differences is given in Table 3. In general, the floor condition introduces smaller changes than the grip position. The difference between the PVC and carpet is in the range of 2° to 3°, while the difference between single arm and both arm holding is in the range of 5° to 10°.

### Motion profile: joint angular velocities

Table 4 shows the median with interquartile range (IQR) as well as the mean maximum and minimum velocity of the joint angular velocities of head/cervical spine, lower back and hip joints.

**Table 4:** Median and interquartile range (IQR) of the joint angle velocities (°/s) C1-Head, T1-C7, T8-S1 cumulative, right and left hip in habitual vacuuming.

Joints	Dimensions	Median velocity (°/s)	IQR velocity (°/s)	Mean maximum velocity (°/s)	Mean minimum velocity (°/s)
C1-Head	Lateral bending	7.16	9.35	22.42	0.18
	Left/right axial rotation	14.77	19.75	39.65	0.32
	Flexion/ extension	6.91	10.03	26.62	0.17
T1-C7	Lateral bending	4.87	6.32	14.36	0.12
	Left/right axial rotation	7.46	9.87	19.94	0.16
	Flexion/ extension	3.78	5.49	14.43	0.09
T9-T8	Lateral bending	1.00	1.36	3.04	0.03
	Left/right axial rotation	2.78	3.15	6.73	0.07
	Flexion/extension	0.70	0.97	2.36	0.02
L1-T12	Lateral bending	1.31	1.78	4.01	0.03
	Left/right axial rotation	3.71	4.21	8.99	0.09
	Flexion/extension	0.94	1.30	3.15	0.02
L4-L3	Lateral bending	1.31	1.78	4.01	0.03
	Left/right axial rotation	3.71	4.21	8.99	0.09
	Flexion/ extension	0.94	1.30	3.15	0.02
L5-S1	Lateral bending	2.41	3.37	8.19	0.06
	Left/right axial rotation	8.55	9.75	20.52	0.21
	Flexion/ extension	2.10	2.89	7.06	0.06
T8-S1 cumulative	Lateral bending	5.88	8.13	18.96	0.14
	Left/right axial rotation	18.74	21.31	45.20	0.45
	Flexion/ extension	4.68	6.46	15.71	0.12
Right hip	Abduction/ adduction	4.69	6.96	15.34	0.12
	Internal/external rotation	7.87	10.46	27.01	0.20
	Flexion/ extension	4.50	7.04	18.09	0.13
Left hip	Abduction/adduction	8.42	11.66	23.31	0.19
	Internal/external rotation	6.43	9.62	27.01	0.16
	Flexion/ extension	6.68	10.86	22.33	0.21

Figure 4a, b shows the frequencies of the angular velocities of the cervical spine during habitual vacuuming. The C1-Head joint shows higher velocities in all three dimensions than the T1-C7 joint.

Figure 5a–e shows the frequencies of angular velocities of the four single trunk joints and T8-S1 cumulative. The velocities in the joints L1–T12 and L4–L3 were identical. In all three dimensions, the velocity increased from T9–T8 to L5–S3. Overall, it can be observed that the angular velocities in the thoracic area are lower compared to the lower trunk and the cervical spine. The C1-Head joint had the highest mean maximum velocities of the spine, while the lowest maximum velocities were observed in T9–T8. Thus, less velocity was observed in the thoracic area than in the cervical and lumbar spine. In all joints of the spine, the highest velocities were in the dimension of rotational movements. On the other hand, the lowest velocities—with only minor differences to lateral flexion—were observed in the dimension of flexion/extension. In rotation, relatively wide IQR could be observed in comparison to flexion/extension and lateral flexion, which indicated a higher individual variability of velocity in this dimension.

Figure 6a, b shows the frequencies of the angular velocities of hip joints. In the movement dimensions of the left hip, the anterior standing leg, relatively high velocities were observed especially in abduction/adduction. In the right hip, the rear standing leg, relatively high velocities were present in rotation. The mean maximum velocities were highest in the left hip.

## Discussion

To our knowledge, this is the first study that performed a sophisticated movement analysis of the trunk and neck based on inertial motion capture in vacuuming. The main findings of this study were that high velocities and movement amplitudes during habitual vacuuming in the spine were observed particularly in the dimension of rotation. The rotation of cervical spine and trunk took place in opposite directions. The movement in the sagittal plane is primarily initiated from the hip joint of the contralateral leg to the dominant hand. Overall, vacuuming was shown to be a dynamic activity without extremely slow or fast angular velocities.

The joint angles of the cervical spine in this study showed certain movement patterns comparable with the studies of Woods and Buckle^5,6^ in professional cleaning staff. Especially subjects cleaning with both arms on the handle can be compared to the ISO standards for a pushing and pulling task (ISO 11228-2): the neck is primarily flexed and rotated during vacuuming. The rotational movements of the cervical spine (C1-Head, T1-C7) were opposite to the rotation in the trunk (T9–T8, L1–T12, L4–L3, L5-S1). At the beginning of the cycle, the cervical spine was slightly turned to the right and then changed to a left rotation towards the middle of the cycle. By contrast, the trunk was rotated to the left at the beginning of the cycle and moved slightly to the right towards the middle of the cycle. The trunk was also primarily held in flexion and was rotated. In the trunk, the difference between maximum and minimum mean over the cycle was very small in flexion/extension, while the movement amplitude in rotation was very pronounced. However, the extent of this movement pattern is still relatively low in habitual vacuuming in this study. Thus, Woods and Buckle^5,6^ could frequently observe trunk flexion angles of 20°–60° and even over 60° when using vacuum cleaners for cleaning activities of professional cleaners. Furthermore, trunk rotations up to 45° were observed^5,6^.

When vacuuming under real conditions, which for instance also requires vacuuming in hard-to-reach places^20^, an intensification of the trunk movement pattern of rotation and flexion posture is conceivable. It should be noted that the aforementioned studies by Woods and Buckle were holistic studies of workplace cleaning equipment, but with rudimentary motion analysis of the actual vacuum cleaning process. In total 10 female professional cleaners were analyzed, while the present study focused on a sophisticated approach in order to capture the cleaning motion of the trunk and neck in detail, reinforced by a sample size of 30 subjects.

Significant differences could be found between several joints between the two floor conditions. Vacuum on PVC leads to joints a bit closer to the neutral position compared to the carpet condition. However, this difference is in general small and is in the range of 2° to 3°. Larger differences of the joint angles could be observed between the holding positions. The difference is especially visible for the rotation at the joint and neck level. During single arm vacuuming the head position is closer to the neutral position (Fig. 1).

In addition, it is noticeable that the flexion of the trunk has wide deviations around the mean movement. Thus, especially the movement in the sagittal plane seems to be influenced by various factors. One factor could be the different vacuum cleaner models. For example, Chang et al.^21^ found significant differences in trunk movements when vacuuming with vacuum cleaners of different canister length. Another factor could be a different anthropometry or different movement habits when vacuuming among the study participants. However, it can be stated that the forward and backward movement of the trunk in the sagittal plane is initiated less by the spinal joints than by the hip joints. Especially in the left hip, the anterior standing leg, there was a clear amplitude in the dimension of flexion/extension compared to the trunk joints. The sagittal movements in the trunk and left hip joint showed that the upper body was primarily in a forward leaning posture when vacuuming. Although the trunk is primarily held in a flexed position, dynamics occur in the rotational movement dimension. When considering all three movement dimensions as a functional unit, there is no pure static trunk work. Furthermore, there were no multidimensional twisted or awkward postures of the trunk. In their review, Wai et al.^13^ referred to higher trunk flexion angles of more than 45° in association with low back pain. The maximum mean in T8-S1 cumulative in flexion was 25.57° ± 9.90°, according to which angles of more than 45° are not achieved in habitual vacuuming in this study. Against this background, there is no risk of low back pain in the habitual vacuuming described here.

The spinal joint angular velocities were lowest in flexion/extension and highest in rotation. Higher velocities were thus shown in dimensions of higher movement amplitudes, such as in the rotation of the spine. It is not surprising that there was less movement dynamics in the thoracic spine than in the cervical and lumbar spine, due to the anatomical structure and the associated limited movement possibilities in the thoracic spine^22^.

It is questionable to what extent the velocities occurring in this study indicate risks for the musculoskeletal system. In the literature, an angular velocity of less than 5°/s for more than 3 s or of more than 90°/s is considered critical for shoulder elevation^23^. The angular velocities in this study were clearly less than 90°/s. Although angular velocities of less than 5°/s were recorded within a cycle, also higher velocities existed over the entire cycle within a cycle duration of 1.65 ± 0.38 s. Accordingly, a critical period of more than 3 s at a velocity of less than 5°/s was not achieved. Only in lateral flexion and flexion/extension of the thoracic and lumbar spine (T9–T8, L1–T12, L4–L3) the velocities were almost exclusively in the range below 5°/s. Hence, it can be stated that vacuuming is rather a dynamic activity which is considered a protective factor for the musculoskeletal system^7^. Yet is has to be questioned, to what extent critical ranges from shoulder elevation^23^ can be transferred to other joints and movement dimensions.

Regarding the risk of musculoskeletal disorders, Gallagher and Heberger^9^ assume no increase but rather a decrease in risk for activities with low force and high repetition, in the risk. Similarly, a clear association of pushing and pulling with low back pain is lacking, as Roffey et al.^12^ describe in their review. Here, they clustered the weight to be pushed and pulled during work activities into classes of < 25 kg, 25–50 kg and > 50 kg. As far as the vacuum cleaners in this study weighed at maximum eight kilogram and the repetition was carried out in a self-chosen speed, this study can at best assume a health-promoting type of vacuuming.

The movement profile of the trunk and neck during habitual vacuuming presented here does not seem to indicate any risks with regard to pushing and pulling or repetitive work, awkward postures or static work sequences. Rather, the movement profile exhibits protective properties. However, it cannot be neglected, that vacuuming, as an everyday activity of cleaning staff, can also have negative effects on the musculoskeletal system, especially the trunk. When vacuuming under real conditions, the cleaning environment can have a crucial influence on the movement behavior—in form of "carrying heavy vacuum" and "moving furniture"^8^—and consequently the musculoskeletal load^5,6,21^. It is possible that the musculoskeletal system is not so much at risk because of the basic movement behavior when vacuuming, but rather because of the movement behavior influenced by the environment.

With regard to this study some limitations have to be considered. On one hand, the leg position and the movement possibilities of the trunk allowed less variability than it would be the case under real conditions. In this respect, the external validity is limited. Wide standard deviations in flexion/extension in the hip joints could, however, be caused by the fact that the width of the step position was not specified. The overall analysis across all conditions showed that there were no risk factors for habitual vacuuming in the described manner. The subjects of this study were healthy and non-professional cleaners. In order to draw conclusions for professional cleaners, it is recommended to include this target group in future studies. Since musculoskeletal complaints, in particular the back and neck, are frequent among cleaning staff, a differentiated comparison of movement patterns between cleaners with complaints and those without can be useful for ergonomics recommendations. Our movement analysis with healthy, non-professional cleaners can be a useful reference in comparison with professional cleaners. Since purely kinematic data were collected in this study, no conclusions about direct clinical implications can be drawn. In future studies, an ergonomic classification of the collected kinematics as well as joint moments calculated via inverse dynamics at relevant body regions (e.g. lower back) could specify the clinical implications of vacuum cleaning.

In summary, it can be said that although we examined a heterogeneous group of subjects (sex, age, height, weight) and these subjects vacuumed on two different surfaces with 8 vacuum cleaners of different construction with one and/or both hands, the mean standard deviation over the vacuuming cycle of the joints of the spine and hip is not greater than 10°. This can be observed in all three directions of movement. As a result, vacuuming in the trunk area is a movement pattern that seems to be the same for everyone.

## Conclusion

The movement patterns of the trunk and neck shown here reflect a comfort range of vacuuming, both in terms of joint angles and joint angular velocity as no extreme values appeared. Small but significant differences in the joint angles exist between different floor conditions (carpet vs. PVC). Large differences could be found in the cleaning positions between using single arms or both arms.

The movement patterns of the trunk and neck shown can thus serve as a reference for working ergonomics in vacuuming. Accordingly, the way the vacuum cleaner is handled creates different demands on the musculoskeletal system.

## Data Availability

All relevant data are in the text.
